# Influence of shaft length on torsional behavior of endodontic nickel–titanium instruments

**DOI:** 10.1007/s10266-020-00572-2

**Published:** 2020-11-27

**Authors:** Gianluca Gambarini, Marco Seracchiani, Alessio Zanza, Gabriele Miccoli, Andrea Del Giudice, Luca Testarelli

**Affiliations:** Department of Oral and Maxillo-Facial Sciences, “La Sapienza” University of Rome, Via Caserta 6, 00161 Rome, Italy

**Keywords:** Endodontics, NiTi files, Shaft length, Torsional stress, TF adaptive

## Abstract

Torsional stresses are one of the most frequent causes of intracanal separation of nickel–titanium endodontic instruments, which represents a great concern of endodontists. For this reason, torsional resistance of rotary instruments has been deeply investigated by determining all parameters that can influenced it, that can be summarized in: (1) Tooth-related factors, (2) Strategy-related factors and (3) Instrument-related factors. This study was conducted to examine the influence of shaft length on torsional resistance of a nickel–titanium rotary instrument and if it should be considered as an Instrument-related factor. With this aim, 120 Twisted Files Adaptive M-L (TFA M-L) NiTi instruments (SybronEndo, Orange, CA, USA) were divided into 6 experimental groups (*n* = *20*), according to instruments length and size: Group 1, 20 TFA M-L1 25/08 23 mm; Group 2, 20 TFA M-L1 25/08 27 mm; Group 3, 20 TFA M-L2 35/06 23 mm; Group 4, 20 TFA M-L2 35/06 27 mm; Group 5, 20 TFA M-L3 50/04 23 mm; and Group 6, 20 TFA M-L3 50/04 27 mm. All instruments were submitted to a static torsional test, blocking each instrument at 3 mm from the tip and rotating it until its fracture. Torque to Fracture (TtF) and fragments length were registered and all data were statistically analyzed. Results showed that Groups 2, 4 and 6 had a higher TtF, respectively, than Groups 1, 3 and 5, which differ from the former just for shaft length. Group 6 showed the highest torsional resistance (1.31 ± 0.08 Ncm), whilst Group 1 the lowest (0.40 ± 0.08 Ncm). According to that, it can be stated that the longer the instrument, the higher the torsional resistance is, proving that shaft length should be considered as an important factor about torsional resistance.

## Introduction

The two main causes of intracanal failure of nickel–titanium (NiTi) rotary instruments are cyclic fatigue and torsional stress [1–3]. Cyclic fatigue occurs when the endodontic instrument is rotated in a curved canal by repeated compressive and tensile stresses, while torsional failure occurs when a part of the instrument, more frequently the tip, binds in the dentine, gets blocked and motor continues to rotate [4–7].

Different strategies can be adopted to reduce the clinical risk of instruments separation for torsional failure. Some of them are related to the clinical use of the NiTi rotary instruments, including applied pressure, pecking motions, increasing coronal flaring, making an efficient glide path or developing motors which allows less stressing movements [8–11].

Other strategies are related to manufacturing process such as metal surface treatments, thermomechanical processing and improvement in the design of endodontic rotary instruments [12, 13]. These features, such as cross-sectional design, pitch, diameter, taper, heat treatments and helix and rake angle, mostly characterize an endodontic nickel–titanium instrument. Despite this, in most cases, some features are standardized, such as the length of the active part of an endodontic instrument, which is 16 mm. However, instruments with different length are available on the marketplace, which are mostly 21, 23, 25, 27, or 31 mm, depending on different brands. This wide range has been thought to adapt instruments to radicular canals length, making endodontic therapies more comfortable. In fact, different lengths should help clinicians to reach apical part of longest teeth, or to make an easier access to the endodontic canals in posterior areas or in case of difficult mouth opening.

Being the active part of an instrument constant, different lengths are obtained by variation of shaft length, that is the non-active part which connects threads to the shank.

As showed by Isik et al., shaft length could influence torsional resistance of an endodontic instruments, due to stress distribution during the instrumentation process; nevertheless, nowadays just above-mentioned study was conducted on the influence of instrument length on the torsional resistance, but none gives an exhaustive physical explanation of this relation [14].

The purpose of this study was to evaluate the influence of shaft length on torsional resistance of a nickel–titanium endodontic instrument, clarifying physical behavior behind this phenomenon.

## Materials and methods

All steps of this study were performed at the Department of Oral and Maxillofacial Sciences of Sapienza University of Rome (Rome, Italy).

120 Twisted Files Adaptive M-L (TFA M-L) NiTi instruments (SybronEndo, Orange, CA, USA) were used for this study. The TFA M-L shaping sequence consists of three NiTi instruments with the following tip sizes and tapers: M-L1 (25.08), M-L2 (35.06) and M-L3 (50.04). They are divided into 6 experimental groups (*n* = *20*), according to instruments length and size:

- Group 1: 20 TFA M-L1 25/08 23 mm

- Group 2: 20 TFA M-L1 25/08 27 mm

- Group 3: 20 TFA M-L2 35/06 23 mm

- Group 4: 20 TFA M-L2 35/06 27 mm

- Group 5: 20 TFA M-L3 50/04 23 mm

- Group 6: 20 TFA M-L3 50/04 27 mm

Before the experiment, every instrument was inspected and measured under a stereomicroscope (Carl Zeiss Micro-imaging, Göttingen, Germany) at 20 × magnification to detect any macroscopic defects and to verify their correct length and none was discarded.

**Fig. 1:**
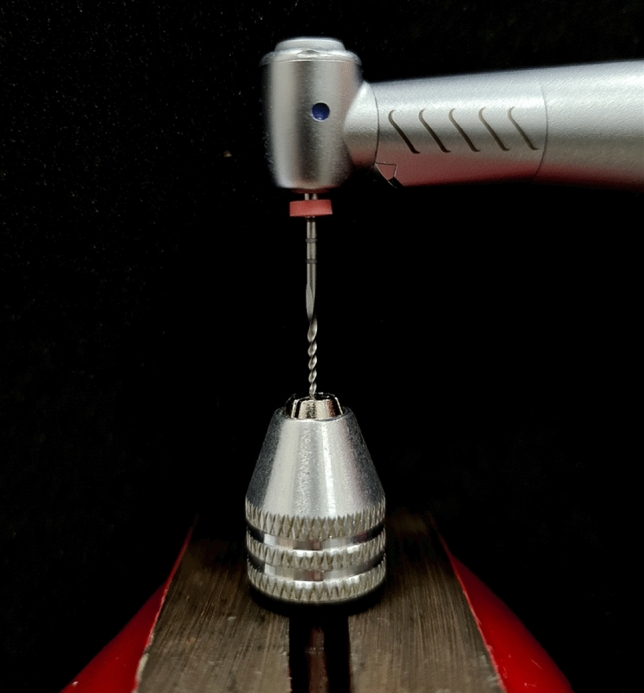
Torsional testing device

All instruments were submitted to a static torsional test, by using a new electric motor validated in recently published manuscript [10]. The device used for static torsional test consists of two parts: a handpiece and a vise used to firmly clench the NiTi instruments. The handpiece is connected to the electric motor (Kavo, Biberach, Germany) allowing a real time (0.1 s) recording of the torque with a precision of 0.05 Ncm. Each instrument was blocked at 3 mm from the tip and it was rotated in a clockwise direction at a constant speed of 300 rpm until its fracture. (Fig. 1).

Torque to fracture (TtF) was registered by the electric motor and fragments were collected and measured with a digital caliper.

All the collected data were analyzed using the Statistical Package for the Social Sciences (IBM‐SPSS, version 23, Shanghai, China). One-way analysis of variance and* t* tests with Bonferroni correction were used to statistically analyze the data at a 5% significance level (*p* < 0.05).

The fractured surface of an instrument, randomly selected from the 120 TF adaptive used in this study, was cleaned in ultrasonic bath and then observed under field emission scanning electron microscope 150x (FE-SEM) (ZEISS Supra 35VP, Oberkochen GmBH, Oberkochen, Germany) to evaluate the pattern of fracture.

## Results

Mean values and standard deviations of TtF obtained from statistical analysis are shown in Table 1. * T* test showed that instruments with a longer shaft length had a higher torsional resistance than same instruments with a shorter one, since the results were statistically significant (*p* value less than 0.05). Also different instruments of the TFA M-L sequence showed a significant difference among them (*p* value less than 0.05), except for TFA M-L1 27 mm and TFA M-L2 23 mm (*p* value more than 0.05).

**Table 1:** TtF (Ncm) according to chosen instruments and different shaft length

	Torque to fracture (Ncm)	
	23 mm	27 mm
TFA M-L1 25/08	0.40 ± 0.08^a^	0.57 ± 0.04^b^
TFA M-L2 35/06	0.63 ± 0.09^b^	0.80 ± 0.07^c^
TFA M-L3 50/04	1.09 ± 0.13^d^	1.31 ± 0.08^e^

Means sharing the same letter are not significantly different (*p* > 0.05)

Mean values and standard deviations of fragments length obtained from statistical analysis are shown in Table 2. *T* test showed a relation statistically not significant between fragment length and shaft length in each comparison (*p* value more than 0.05).

**Table 2:** Fragments’ length (mm) according to chosen instruments and different shaft length

	Fragments’ length (mm)	
	23 mm	27 mm
TFA M-L1 25/08	2.90 ± 0.39^a^	3.02 ± 0.22^a^
TFA M-L2 35/06	3.42 ± 0.26^a^	3.48 ± 0.13^a^
TFA M-L3 50/04	3.09 ± 0.35^a^	3.28 ± 0.21^a^

Means sharing the same letter are not significantly different (*p* > 0.05)

**Fig. 2:**
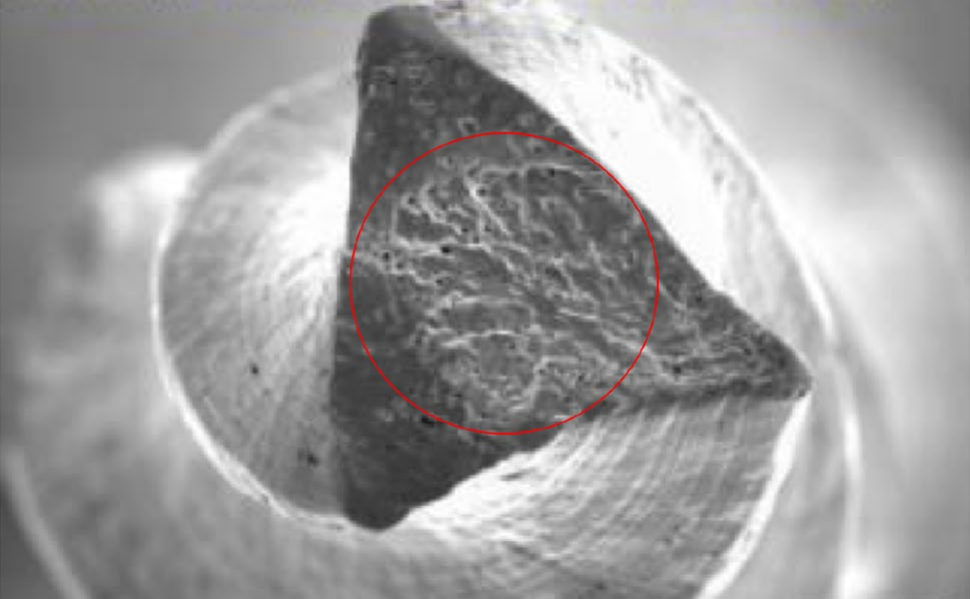
FE-SEM of fractured surface of a TF adaptive instrument after torsional fracture. This cross-sectional view showed typical features of torsional failure as multiple skewed dimples and circular abrasion marks (red circle)

FE-SEM image of the fractured surface of the instrument randomly selected showed the typical features of torsional failure: skewed dimples near the center of rotation (Fig. 2).

## Discussion

Since the introduction of NiTi instruments, endodontic clinical practice had become faster, more precise, easier, ensuring more predictable results increasing mechanical and chemical bacterial removal. Despite this, intracanal separation of instruments has remained one of the most concern for endodontics clinicians [15, 16].

The two main causes of intracanal separation are undoubtedly cyclic fatigue and torsional failure. In literature, there is a heterogeneous view about the prevalence of one cause rather than another one, but certainly both can be considered essential in determining safe use of instruments [17]. Torsional strength indicates the improved performance of instrument to twist before fracture during canal instrumentation.

Over the years, torsional resistance of endodontic rotary instruments has been deeply investigated by determining all parameters that can influenced it. They can be divided into:

1. Tooth-related factors

2. Strategy-related factors

3. Instrument-related factors

The first group contains all characteristic of tooth that can influence generation of torsional stresses, such as canal diameter, canal curvature, dentin hardness and canal length [18–20]. On the other hand, the second group consists of all parameters that characterize clinical approach such as access cavity, coronal preflaring, glide path, high or low torque control motor, selected technique and used motions as brushing motion, pecking motion, continues or reciprocating motion [10, 11, 17, 21–24]. Finally, the last group is composed of all features that characterize endodontic instruments. The most remarkable are cross-sectional design, type of alloy, manufacturing process, heat treatment, inner core area, pitch, helix and rake angle, size diameter and taper [17, 25–27]. According to the results of this study, it is reasonable to consider shaft length as an instrument-related factor that can influence torsional resistance.

The rationale behind the choice of manufacturers to fabricate instruments of different length is to facilitate endodontic practice in all situations that require a shorter or a longer instrument, such as difficulties in mouth opening, treatments in posterior area, treatments in children or the presence of a tooth with long roots. The active part of an endodontic instrument is mostly standardized to 16 mm; for this reason, different lengths reside in different shaft lengths and most common are 18 mm, 21 mm, 23 mm, 25 mm, 27 mm and 31 mm, depending on different brands. However, the results of the present study suggest that the choice to use an instrument with a defined length influences its torsional behavior.

In this study, the three components of Twisted Files Adaptive M-L sequence (SybronEndo, Orange, CA, USA) with different lengths were compared in a torsional static test to evaluate the influence of shaft length on torsional resistance and fragments’ lengths.

Twisted File Adaptive instruments are characterized by an equilateral triangular cross-section design and they are manufactured by a proper technique of heating and cooling that causes an R-phase molecular structure, in which the alloy can be subjected to twist, increasing flexural properties of these instruments [28, 29]. However, this R-phase NiTi alloy and its manufacturer process decrease torsional resistance comparing to other NiTi instruments [30]. Twisted File Adaptive instruments are available just in two different lengths, 23 mm and 27 mm.

On the basis of our results, group 6 (TFA M-L3 50/04 27 mm) had the best torsional resistance in term of TtF. This result is in accordance with other studies present in literature that show the relevance of central mass and diameter at the point where maximum stress is applied (3 mm from the tip) concerning torsional resistance tests [28, 30]. This concept is confirmed by the other two results regarding group 4 (TFA M-L2 35/06 27 mm) and group 2 (TFA M-L1 25/08 27 mm), that show the greater torsional resistance of group 4 compared to group 2. The same is considered for the TFA M-L sequence with 23 mm instruments length. Therefore, the above-mentioned results confirm the relevance of cross-sectional mass on torsional resistance of NiTi rotary files.

On the other hand, the comparison of Torque to Fracture between group 2 (TFA M-L1 25/08 27 mm) and group 3 (TFA M-L2 35/06 23 mm) showed no statistically significant difference, as shown in Table 1. Therefore, resistance to torsional stresses seems to be equal for these instruments. Since torsional resistance depends mostly on cross-sectional mass and diameter at the point of fracture, this result may seem to be unexpected. Anyway, the difference in cross-sectional mass seems to be compensated by different length of the two instruments, respectively, 27 mm for TFA M-L1 and 23 mm for TFA M-L2, and in particular their shaft length. It is reasonable to think that shaft length, in case of equal active part of instruments, is a parameter that directly influences torsional resistance in the same way of cross-sectional mass, diameter and all other instrument-related factors. This hypothesis seems to be confirmed by the differences in torque resistance evidenced comparing group 1 and group 2, group 3 and group 4, group 5 and group 6. Indeed, since these results are statistically significant, we can assume that increasing the shaft length, increases the torsional resistance as well. This finding is in accordance with the only study, at the moment, present in literature that evaluates this relation, affirming that a longer shaft length might result in higher torsional strength and toughness [14]. Anyway, there are no studies that deeply explain, in physical terms, the rationale behind this phenomenon.

**Fig. 3:**
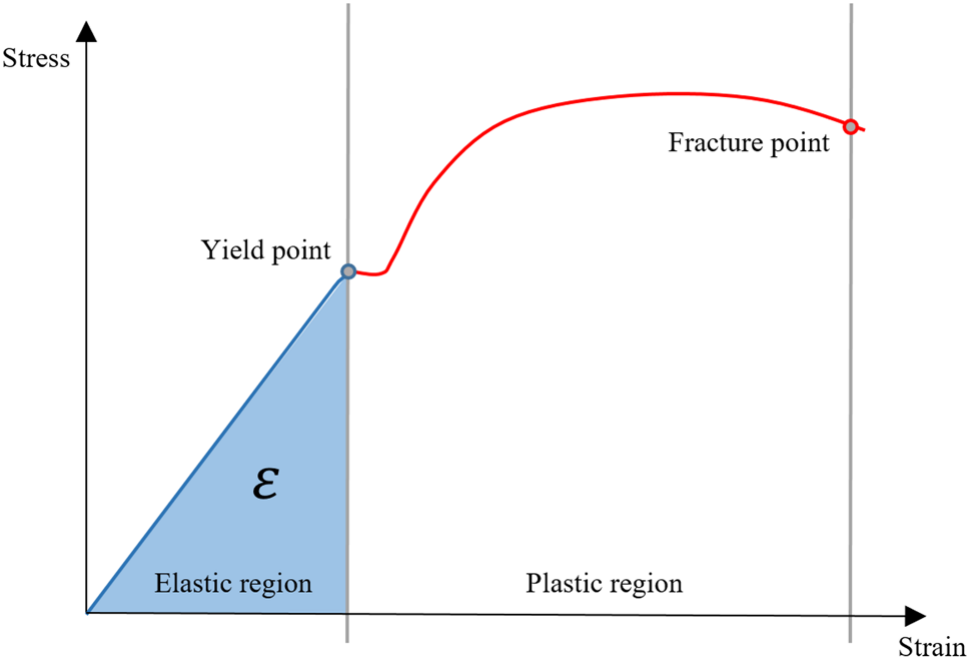
Schematic stress–strain curve showing torsional absorbed energy ($\varepsilon$, blue area)

The physical basis behind the increased torsional resistance for longer instruments can be found in the concept of torsional limit and torsional absorbed energy. Torsional limit can be considered as the threshold beyond which an increase in torsional stress leads to plastic deformation or fracture of the instrument (yield point for torsional load), while torsional absorbed energy can be understood as the ability of an instrument to absorb energy before its plastic deformation, remaining in the elastic region of the stress–strain curve [31] (Fig. 3).

**Fig. 4:**
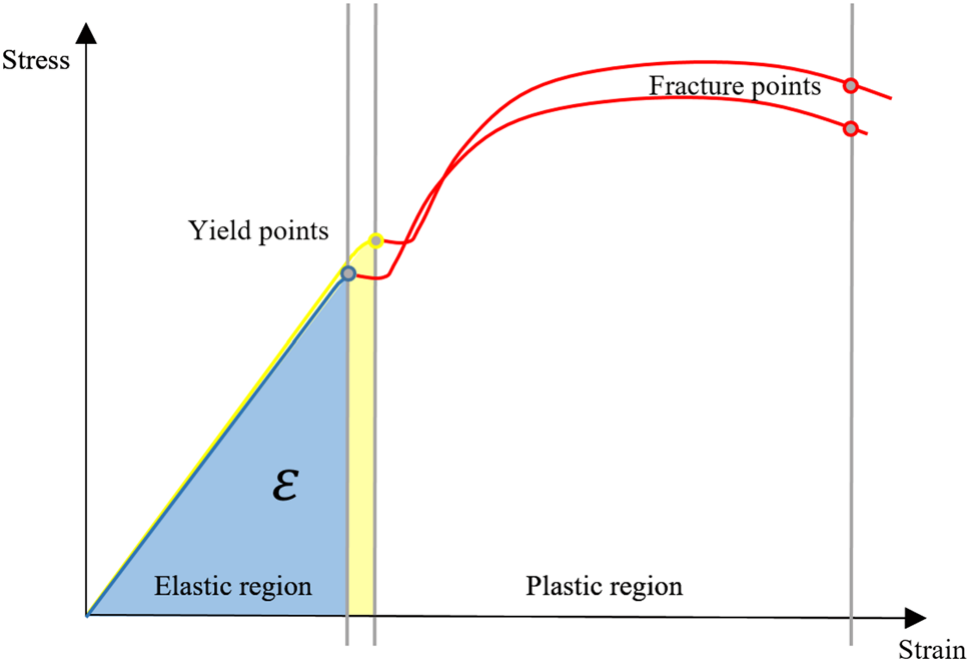
Schematic diagram showing the different ability of a longer and a shorter instrument to absorb energy without plastic deformation. Yellow area is the torsional absorbed energy $(\varepsilon )$ of a longer instrument, whilst the blue is the torsional absorbed energy $(\varepsilon )$ of a shorter instrument

Mathematically, considering stress–strain curve of NiTi (Fig. 2), the torsional absorbed energy is the area under elastic curve that ends in correspondence to the torsional limit value (Yield point for torsional load) and it can be defined with the following simplified formula:

$$\varepsilon \cong \frac{1}{2}\mathop \int \limits_{0}^{l} G \cdot I_{P} \cdot {\upgamma }^{2} \cdot {\text{d}}x,$$

where $\varepsilon$ is the stored energy, $G$ is tangential modulus of elasticity,$I_{P}$ is the polar moment of Inertia, that depends on the cross-sectional shape, γ^2^⋅ⅆx is the square of the angular deformation of each section of the instrument and $l$ is the length of the instrument. As demonstrated by this formula, torsional stored energy depends on the integral from 0 to $l$, for this reason increasing shaft length, being the active part of an instrument constant, $\varepsilon$ increases. Increasing $\varepsilon$, the torsional limit value increases (on stress–strain curve of NiTi, its point is shifted towards higher values of strain) and consequently torsional resistance increases [32]. In other words, a longer instrument is able to absorb more energy before its plastic deformation than a shorter instrument (Fig. 4).

## Conclusion

Upon the results, it can be affirmed that the longer the instruments, the higher its resistance to torsional stresses is. According to that, during instrumentation of narrow canals or in case of high risk for torsional failure, patient’s conditions permitting, it should be recommended to use a longer instrument to increase its torsional resistance and to reduce the likelihood of instrument separation.
